# Maternal and Perinatal Outcomes among Maternity Waiting Home Users and Non-Users in Rural Rwanda

**DOI:** 10.3390/ijerph182111211

**Published:** 2021-10-26

**Authors:** Edwin Tayebwa, Richard Kalisa, Amedee Fidele Ndibaza, Lisa Cornelissen, Eefje Klein Teeselink, Young-Mi Kim, Jeroen van Dillen, Jelle Stekelenburg

**Affiliations:** 1 University Medical Centre Groningen, Department of Health Sciences, Global Health, University of Groningen, 9700 RB Groningen, The Netherlands; jelle.stekelenburg@online.nl; 2 IntraHealth International, Kigali 6639, Rwanda; amedee.ndibaza@gmail.com; 3 School of Public Health, University of Rwanda, Kigali 3286, Rwanda; 4 Gelre Hospital, Albert Schweitzerlaan 31, 7334 DZ Apeldoorn, The Netherlands; lisacornelissen57@gmail.com; 5 Amalia Children’s Hospital, Radboudumc Nijmegen, 6500 HB Nijmegen, The Netherlands; eefje.kleinteeselink@radboudumc.nl (E.K.T.); jeroen.vandillen1@radboudumc.nl (J.v.D.); 6 Jhpiego, Johns Hopkins University, Baltimore, MD 21231, USA; Young-Mi.Kim@jhpiego.org; 7 Department of Obstetrics and Gynaecology, Leeuwarden Medical Centre, 8934 AD Leeuwarden, The Netherlands

**Keywords:** maternity waiting home, maternal mortality, maternal morbidity, stillbirth, prior caesarean section

## Abstract

Most maternal and perinatal deaths could be prevented through timely access to skilled birth attendants. Women should access appropriate obstetric care during pregnancy, labor, and puerperium. Maternity waiting homes (MWHs) permit access to emergency obstetric care when labor starts. This study compared maternal and perinatal outcomes among MWH users and non-users through a retrospective cohort study. Data were collected through obstetric chart reviews and analyzed using STATA version 15. Of the 8144 deliveries reported between 2015 and 2019, 1305 women had high-risk pregnancies and were included in the study. MWH users had more spontaneous vaginal deliveries compared to non-users (38.6% versus 16.8%) and less cesarean sections (57.7% versus 76.7%). Maternal morbidities such as postpartum hemorrhage occurred less frequently among users than non-users (2.13% versus 5.64%). Four women died among non-users while there was no death among users. Non-users had more stillbirths than users (7.68% versus 0.91%). The MWH may have contributed to the observed differences in outcomes. However, many women with high risk pregnancies did not use the MWH, indicating a probable gap in awareness, usefulness, or their inability to stay due to other responsibilities at home. Use of MWHs at scale could improve maternal and perinatal outcomes in Rwanda.

## 1. Introduction

Globally, 295,000 maternal deaths and 2.6 million stillbirths were estimated to have occurred in 2017, and the majority of these could have been prevented through timely access to skilled birth attendants [1,2,3]. Many of the maternal deaths have been attributed to barriers in access like unfavorable geographical terrain, lack of transport/communication, long travel times due to poor roads, cultural beliefs, high costs, and reluctance to move and seek care before labor [4,5,6,7]. To make pregnancy and birth safer, every woman should have access to appropriate obstetric care during pregnancy, delivery, and puerperium [7].

Over the last 20 years, Rwanda has made remarkable reductions in maternal deaths from 1071 to 203 per 100,000 live births [8,9]. The most common causes of maternal death are postpartum hemorrhage (PPH), obstructed labor, puerperal sepsis, eclampsia, and unsafe abortion [10,11]. Of these cases, more than 60% were a result of suboptimal care [11]. Thus, these maternal deaths could have been prevented with provision of quality antenatal care (ANC), intrapartum care, equitable distribution of emergency obstetric and newborn care facilities, strengthening the referral system, and increasing access to quality maternity services [6,7,11]. One of the strategies for increasing access and reducing maternal morbidity and mortality is the implementation of maternity waiting homes (MWHs) [6,7,12].

Maternity waiting homes are dedicated places where high-risk pregnant women are provided with accommodation during their final weeks of pregnancy, allowing them easy access to emergency obstetric care when labor starts. Upon admission, pregnant women are provided with health education about safe pregnancy, labor, and newborn care [13]. The WHO recommends establishment of MWHs close to a health facility where essential care for childbirth is provided, especially targeting women living in remote areas [13]. Over the last few decades, developing countries have been scaling up MWHs to bridge geographical barriers between health facilities and communities, with promising results in reducing maternal and neonatal morbidity and mortality [14,15,16,17]. A Cochrane review in 2012 found insufficient evidence on the potential benefit of MWHs (6). However, several recent studies seem to underline that MWHs improve maternal and neonatal mortality [13,14,15], though their use, services offered, and management standards differed greatly between countries [14,15,16,17].

In Rwanda, distance to health facilities has been reported as the second most important barrier for women to access obstetric care [10,11]. We aimed to understand the impact of a MWH on maternal and perinatal outcomes among high-risk pregnant women in a rural district in Rwanda.

## 2. Materials and Methods

The study was conducted at Ruli Hospital (RH) in Gakenke District, Rwanda. Geographically, Gakenke District has a mountainous landscape with an average altitude of 1788 m above sea level. According to the Rwanda Health Management Information System, the district’s maternal mortality ratio and skilled birth attendance were 325 per 100,000 live births and 96.7%, respectively by December 2019. About 50% of households walk for more than 1 h to reach a nearby health facility. Ruli Hospital serves a population of about 110,548 inhabitants (projections from the 2012 census) and also receives patients from neighboring districts. The hospital has a bed capacity of 179 and provides a wide range of services including comprehensive emergency obstetric and newborn care for eight health centers and eight health posts within their catchment area. During the study period, the medical staff in the maternity ward consisted of one medical officer, five nurses, and nine midwives. There was no obstetrician. Furthermore, the hospital has an operating theatre, a laboratory, a medical imaging unit, and blood transfusion services.

In 2011, *Matres Mundi*, an international non-governmental organization, supported RH to set up a MWH within the hospital premises, which by the end of 2019 had received over 700 pregnant women. It is the only MWH in Rwanda. While staying in the MWH, pregnant women received obstetric care; psycho-social care; peer support; and education on proper nutrition, breastfeeding, and birth preparedness. Additionally, they were taught hands-on skills such as gardening, cooking, making handcrafts, and knitting baby items. All medical services offered are paid for by the community-based health insurance scheme (commonly known as “*mutuelle de sante*”) and pregnant women contributed 10% of the cost.

A facility-based retrospective cohort study was conducted. Over a period of 5 years (January 2015 to December 2019), 8144 women gave birth in RH. Among those, 1305 women were eligible for staying in the MWH while 6839 women were not eligible. Eligibility for admission to the MWH was determined by a medical officer on duty based on predefined criteria that consisted of the following: a problem related to the pregnancy e.g., history of an abortion; a caesarian section (CS); prolonged labor, as well as problems during the current pregnancy, including preterm premature rupture of membranes, antepartum hemorrhage, reduced fetal movement, pre-eclampsia, etc. and one of the following conditions: at least 36 weeks of amenorrhea, place of residence that was far from the hospital (more than 3 h of walking), having no one to take care of the woman at home, victim of gender-based violence, being in social economic category 1 or 2, as well as clinician decision.

**Figure 1:**
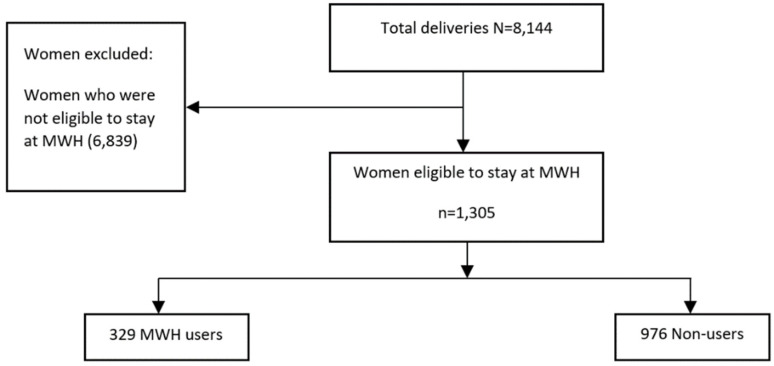
Inclusion and exclusion of users and non-users.

Among the 1305 eligible women, 329 MWH-users (women who delivered after having stayed at the MWH) and 976 non-users (women who delivered but had not stayed at the MWH) were compared on maternal and perinatal outcomes. Non-users were identified based on the assumption that they met the same admission criteria as users (see Table 1). We identified MWH users and non-users by following the elimination process as shown in Figure 1.

Data were collected from hard copy medical records by two trained research assistants using a pre-designed data collection form (see Supplementary File, Figure S1) that was designed using Kobo Toolbox (version 2018) and installed on Android tablets under the supervision of the principal investigator (ET). Information from files included sociodemographic characteristics, ANC, indication of MWH and non-MWH admissions, obstetric history, mode of delivery, complications during delivery, outcome of delivery (maternal and perinatal), etc., which were abstracted from obstetric charts. Data were exported from Kobo Toolbox to MS Excel 2016 and then to STATA 15 for cleaning and analysis. Demographic characteristics were compared between MWH users and non-users using chi-square statistic. Univariable logistic analysis was conducted to determine associations between dependent and independent variables. Crude Odds Ratios (cOR) and their 95% confidence intervals (CI) were calculated, and significant outcomes with *p* value < 0.05 were considered for multivariable logistic regression. Adjusted Odds Ratios (aOR) and their 95% CI were calculated to take care of potential confounding and identify variables that showed statistically significant differences in the two groups. The ORs were adjusted for age, parity, occupation, and ownership of health insurance. Ethical clearance was obtained from the Rwanda National Ethics Committee (protocol code 335/RNEC/2020).

## 3. Results

### 3.1. Indications for Admission

Based on the admission criteria for the MWH that were set by the hospital, results showed that there were many women who qualified to stay at the MWH but never did, but came to the hospital when they were going to deliver instead. The main indication for staying in the MWH for those who used it, and among women who met the criteria for admission to the MWH but did not use it, was previous CS (Table 1).

### 3.2. Socio-Demographic Characteristics of Women (MWH-Users and Non-Users)

Overall, most women were married (75.6%), were subsistence farmers (97.4%), and had health insurance (99.5%). The mean age of the study population was 29 years and 30 years for MWH users and non-users respectively. Both groups of women did not have statistically significant differences in occupation, ownership of health insurance, parity, ANC attendance, and having a history of prenatal maternal disease. However, the following variables were significantly different between users and non-users: maternal age (≤25 years), marital status (being single), having a prior stillbirth, and having a previous CS (Table 2).

### 3.3. Maternal and Perinatal Outcomes

Maternal and perinatal outcomes among MWH-users and non-users are shown in Table 3. Spontaneous vaginal delivery was a more common mode of delivery among MWH-users (127/329; [38.6%]) compared to non-users (164/976; [16.8%]), while there were more deliveries by CS among non-users (749/976; [76.74%]) compared to users (190/329; [57.75%]). Analysis showed that the odds of MWH-users delivering by a CS was reduced (crude odds ratio [cOR] 0.33, 95% CI 0.25–0.43) and were also less likely to require vacuum extraction at delivery (cOR 0.25, 95%CI 0.13–0.46). MWH-users had less chances of developing PPH after delivery. Although not statistically significant, fewer MWH-users developed puerperal sepsis (*p* = 0.149). Results indicate that there were no maternal deaths reported among MWH-users, as opposed to four maternal deaths among non-users, equivalent to 410 per 100,000 live births. The causes of maternal death were associated with pulmonary embolism (n = 2) and uterine rupture (n = 2). In addition, MWH-users had less stillbirths (cOR 0.11, 95% CI 0.04–0.35). After delivery, the occurrence of perinatal asphyxia was more common among newborns whose mothers had not used MWH (4.9%) compared to those whose mothers had used the MWH (3.1%), although there was no statistically significant difference between the two groups (*p* = 0.157). There was a significant difference between MWH-users and non-users in the number of neonatal deaths (OR 0.11, 95% CI 0.14–0.35), indicating an association with use of the MWH, where MWH-users had reduced odds of having neonatal deaths.

Multivariable analysis showed that there was a significant association between use of the MWH and delivery by CS (aOR 0.24, 95% CI 0.18–0.33) as well as use of the MWH and delivery by vacuum extraction (aOR 0.20, 95% CI 0.10–0.40), see Table 4. There was an increased chance of having a stillbirth for women who did not use the MWH (aOR 0.05, 95% CI 0.02 to 0.16). After adjusting for confounders such as age, parity, occupation, and ownership of health insurance, birth asphyxia and stillbirth were also associated with use of MWH (aOR 0.46, 95% CI 0.22-0.98 and aOR 0.05, 95% CI 0.02–0.16 respectively).

## 4. Discussion

This is the first study on MWHs in Rwanda, and it contributes to the existing literature from low-income settings. The MWH at RH receives victims of gender-based violence, mothers from very poor backgrounds based on *Ubudehe* (collective action to combat poverty) category 1 and 2 according to the national categorization, mothers who live far away from the hospital, as well as those with (high risk of) obstetric complications [18]. The majority of women in the study were subsistence farmers and had medical insurance. The findings showed statistically significant differences between MWH-users and non-users as it relates to prior stillbirths and having a previous CS. Additionally, the MWH users and non-users differed by maternal age (≤25 years) and marital status (being single), which is similar to results from other studies in other countries [6,15,16,17], although little is known about these factors in Rwanda [12,19,20]. This is in keeping with what was observed at the MWH during data collection where most mothers admitted at the MWH were young and included single mothers. The observed differences were not expected especially since both MWH-users and non-users did not differ by ANC attendance and having medical insurance that pays for MWH services among others. The proportion of non-users meeting the criteria for admission to the MWH based on prior CS (40%) is higher than those who had prior CS among MWH-users (26.4%). This raises a question of whether the community or ANC providers (at other health facilities in the district) knew about the existence and/or benefits of the MWH at RH. Since this is the only MWH in the country, it may be presumed that many people do not know about it and, therefore, there is a need to educate mothers and providers on benefits of the MWH during routine ANC. It is also possible that the mothers knew about the MWH but did not stay there because of other family responsibilities at their homes.

The mode of delivery, as part of maternal outcomes for this study, was significantly different between MWH-users and non-users, where the number of CS and use of vacuum extraction were significantly lower among MWH-users compared to non-users. These results are different from what was observed in another study in Ethiopia where MWH-users were more likely to undergo a CS [21]. Although we did not analyze the length of stay at the MWH, we assume that MWH-users at RH had time to rest before delivery and received regular obstetric care while in the MWH. Another reason could be that the non-users had to travel to the hospital at onset of labor and arrived when it was too late to consider use of conservative means of delivery. In addition, the proportion of non-users with history of CS was already higher than that of MWH-users. It is important to note that due to inadequate staffing to monitor several women in labor at once, the hospital does not encourage trial of labor for women with a history of CS. There is need to equip midwives and medical officers with knowledge and skills to perform vacuum deliveries to minimize unnecessary CS. This demonstrates the need for women with high-risk pregnancies to use MWHs, hence the need to scale up MWH to other districts with similar rural settings.

The study found differences in the occurrence of PPH between MWH-users and non-users, which is consistent with findings from other authors [5,17,22] that showed that MWH-users had less maternal complications and were less likely to die. In terms of perinatal outcomes, MWH-users had fewer birth asphyxia cases (3%) compared to non-users (4.9%), which was significantly different. We found a significant difference in fresh stillbirth and immediate neonatal death where MWH-users were less likely to have fresh stillbirths or lose their newborns. Similar reductions in stillbirth and neonatal death among MWH-users were reported in previous studies conducted in Ethiopia [5,14,16,21,22] as well as a recent systematic review [15]. During the study period, there were no maternal deaths among MWH-users while there were four maternal deaths among the non-users group, equivalent to 410 per 100,000 live births, which is approximately twice as high as the national maternal mortality rate [9]. Overall, it is important to note that women in the MWH at RH benefit from regular obstetric monitoring through visits by medical doctors, midwives, and nurses. They also receive psycho-social support through daily sessions with social workers. Consequently, mothers who stay at this MWH were less likely to have birth complications despite their high-risk pregnancies, and this could be due to that fact they were closer to trained health care workers who are able to provide emergency obstetric care at onset of labor. This stresses the need for early identification, referral, and monitoring of women with high-risk pregnancies. In addition, to address challenges faced by rural women in seeking or reaching care [11], it seems necessary to encourage women with uterine scars and other obstetric complications to use the MWH [1,5,16]. Scaling up the MWH to other districts might help to improve maternal and perinatal outcomes.

The retrospective nature of this study had inherent limitations because of its reliance on routinely collected obstetric data in patient charts, which may have missing information. The study team extended the study period to 5 years to compensate for this limitation. Despite the limitation, our findings provide evidence that the use of the MWH at RH contributed to improving maternal and perinatal outcomes, and that mothers admitted in the MWH receive vital services that they would otherwise not have accessed. However, we did not study the quality of care provided to the mothers during their stay at the MWH. Therefore, an audit of the quality of care provided to women in the MWH would shed more light on this. In addition, since a large proportion of women with indications for admission to the MWH did not use it, further studies would be needed to evaluate why women do not use the MWH.

## 5. Conclusions

There is evidence that use of the MWH at RH was associated with improved maternal and perinatal outcomes among mothers with high-risk pregnancies. Mothers who used the MWH had better birth outcomes. However, a large proportion of women with indications for admission to the MWH did not use it. This indicates a probable gap in awareness about the existence and usefulness of the MWH as well as a possible inability of the mothers to stay at the MWH due to family responsibilities. Use of MWHs at scale could further improve maternal and perinatal outcomes in Rwanda.

## Figures and Tables

**Table 1:** Indications for admission to the MWH.

Indications	Users, *n* = 329 (%)	Non-Users, *n* = 976 (%)	Total, *n* = 1305
Previous CS	87 (26.4)	390 (40.0)	477
Post term pregnancy	21 (6.4)	58 (5.9)	79
PPROM *	6 (1.8)	49 (5.0)	55
Abdominal pain/lumbopelvic pain	20 (6.1)	32 (3.3)	52
Preterm labor	15 (4.6)	34 (3.5)	49
Pre-eclampsia	8 (2.4)	32 (3.3)	40
Excessive fundal height	6 (1.8)	32 (3.3)	38
Fetal abnormal lie/presentation	11 (3.3)	32 (3.3)	43
CPD **	6 (1.8)	32 (3.3)	38
Suspected IUFD ***	7 (2.1)	19 (1.9)	26
History of prolonged labor	4 (1.2)	22 (2.3)	26
Fetal distress	7 (2.1)	31 (3.2)	38
Other	97 (29.5)	213 (21.8)	310
Not documented	34 (10.3)	0 (0.0)	34

* preterm premature rupture of membranes, ** cephalopelvic disproportion, *** intrauterine fetal death.

**Table 2:** Characteristics of women (MWH-users versus non-users).

Characteristics	MWH Users		MWH Non-Users		Total	X^2^	*p*-Value
	*n* = 329	%	*n* = 976	%	*n* = 1305		
**Maternal age**							
≤25	121	36.78	268	27.46	389	10.2	0.006
26–30	78	23.71	270	27.66	348		
≥31	130	39.51	438	44.88	568		
**Parity**							
P0	106	32.22	295	30.23	401	2.5	0.285
P1–P3	202	61.4	637	65.27	839		
P4 and above	21	6.38	44	4.51	65		
**Marital status**							
Married	224	68.09	762	78.07	986	23.6	0.000
Single	73	22.19	114	11.68	187		
Co-habiting	4	1.22	13	1.33	17		
Separated/Divorced	3	0.91	4	0.41	7		
Not documented	25	7.6	83	8.5	108		
**Occupation**							
Formal/Salaried employee	4	1.22	19	1.95	23	3.1	0.364
Subsistence farmer	321	97.57	950	97.54	1271		
Business	4	1.22	5	0.51	9		
Not documented	0	0.0	2	0.20	2		
**Health Insurance**							
Yes	327	99.39	971	99.49	1298	0.04	0.837
No	2	0.61	5	0.51	7		
**Prior stillbirth**							
Yes	5	1.52	28	2.87	33	13.7	0.001
No	323	98.18	908	93.03	1231		
Not documented	1	0.30	40	4.10	41		
Number of ANC * visits							
ANC < 4	92	27.96	236	24.18	328	4.1	0.127
ANC ≥ 4	55	16.71	209	21.41	264		
Not documented	182	55.32	531	54.4	713		
**History of prenatal maternal disease**							
Yes	41	12.46	133	13.63	174	2.7	0.245
No	287	87.23	830	85.04	1117		
Not documented	1	0.3	13	1.33	14		
Prior C/S **							
Yes	128	38.91	463	47.44	591	7.2	0.007
No	201	61.09	513	52.56	714		

* ANC = antenatal care, ** Prior C/S = prior caesarean section.

**Table 3:** Odds ratios for maternal and perinatal outcomes between users and non-users.

Outcomes	MWH Users *n* = 329 (%)	MWH Non-Users *n* = 976 (%)	OR (95% CI)	*p*-Value
**Maternal**				
**Mode of delivery**				
Spontaneous vaginal delivery	127 (38.6)	164 (16.8)	(Ref)	(Ref)
Cesarean section	190 (57.75)	749 (76.74)	0.33 (0.25–0.43)	0.000
Vacuum extraction	12 (3.65)	63 (6.45)	0.25 (0.13–0.46)	0.000
**Type of maternal complication**				
No maternal complication	321 (97.6)	854 (87.4)	(Ref)	(Ref)
PPH	7 (2.13)	55 (5.64)	0.33 (0.15–0.75)	0.008
Eclampsia	0 (0.0)	4 (0.41)	-	-
Severe pre-eclampsia	0 (0.0)	3 (0.31)	-	-
Puerperal sepsis	1 (0.3)	12 (1.23)	0.22 (0.02–1.71)	0.149
Ruptured uterus	0 (0)	9 (0.92)	-	-
Others	0 (0.0)	39 (4.0)	-	-
**Maternal outcome**				
Live	329 (100)	972 (99.59)	(Ref)	(Ref)
Death	0 (0.0)	4 (0.41)	-	-
**Perinatal**				
**Birth Asphyxia**				
No	319 (96.9)	928 (95.1)	(Ref)	(Ref)
Yes	10 (3.1)	48 (4.9)	0.6 (0.3–1.21)	0.157
**Immediate neonatal outcome**				
Live birth	326 (99.09)	901 (92.32)	(Ref)	(Ref)
Stillbirth	3 (0.91)	75 (7.68)	0.11 (0.04–0.35)	0.000
**Type of stillbirth**				
Fresh	2 (66.67)	41 (54.67)	(Ref)	(Ref)
Macerated	0 (0)	22 (29.33)	_	_
Not documented	1 (33.33)	12 (16)	1.7 (0.14–20.5)	0.673
**Neonatal outcomes**				
Discharged	325 (99.69)	898 (99.67)	(Ref)	(Ref)
Died	1 (0.31)	3 (0.33)	0.92 (0.1–8.9)	0.943
Referred	3 (0.91)	75 (7.68)	0.11 (0.03–0.35)	0.000

**Table 4:** Adjusted odds ratios for maternal and perinatal outcomes between users and non-users.

Outcomes	MWH Users *n* = 329 (%)	MWH Non-Users *n* = 976 (%)	aOR (95% CI)	*p*-Value
**Maternal**				
**Mode of delivery**				
Spontaneous vaginal delivery	127 (38.6)	164 (16.8)	(Ref)	(Ref)
Cesarean section	190 (57.75)	749 (76.74)	0.24 (0.18–0.33)	0.000
Vacuum extraction	12 (3.65)	63 (6.45)	0.20 (0.10–0.40)	0.000
**Perinatal**				
**Birth Asphyxia**				
No	319 (96.9)	928 (95.1)	(Ref)	(Ref)
Yes	10 (3.1)	48 (4.9)	0.46 (0.22–0.98)	0.044
**Immediate neonatal outcome**				
Live birth	326 (99.09)	901 (92.32)	(Ref)	(Ref)
Stillbirth	3 (0.91)	75 (7.68)	0.05 (0.02–0.16)	0.000

## Data Availability

Data may be provided by the principal investigator upon request.

## Supplementary Materials

The following are available online at https://www.mdpi.com/article/10.3390/ijerph182111211/s1, Figure S1: data collection tool.
