# Supplementary material for: Maternal and Perinatal Outcomes among Maternity Waiting Home Users and Non-Users in Rural Rwanda
Source: Int J Environ Res Public Health. 2021 Oct 26;18(21):11211. doi: 10.3390/ijerph182111211 (PMC8583170; doi:10.3390/ijerph182111211)
Supplement: Supplementary file 1 [file ijerph-18-11211-s001.zip › ijerph-1363917-supplementary.pdf]

https://ee.kobotoolbox.org/x/zm5HWM6y
